# Evaluation of a Novel Oncolytic Adenovirus Silencing *SYVN1*

**DOI:** 10.3390/ijms232315430

**Published:** 2022-12-06

**Authors:** Christie Vermeulen, Tereza Brachtlova, Nikki Tol, Ida H. van der Meulen-Muileman, Jasmina Hodzic, Henri J. van de Vrugt, Victor W. van Beusechem

**Affiliations:** 1 Amsterdam UMC location Vrije Universiteit Amsterdam, Medical Oncology, De Boelelaan 1117, 1081 HV Amsterdam, The Netherlands; 2 ORCA Therapeutics BV, Onderwijsboulevard 225, 5223 DE ‘s Hertogenbosch, The Netherlands; 3 Cancer Center Amsterdam, Cancer Biology and Immunology, Amsterdam, The Netherlands; 4 Amsterdam Institute for Infection and Immunity, Cancer Immunology, Amsterdam, The Netherlands; 5 Amsterdam UMC location Vrije Universiteit Amsterdam, Human Genetics, De Boelelaan 1117, 1081 HV Amsterdam, The Netherlands

**Keywords:** oncolytic adenovirus, RNA interference, TP53, SYVN1/HRD1/DER3, cancer-cell-killing potency

## Abstract

Oncolytic adenoviruses are promising new anticancer agents. To realize their full anticancer potential, they are being engineered to express therapeutic payloads. Tumor suppressor p53 function contributes to oncolytic adenovirus activity. Many cancer cells carry an intact *TP53* gene but express p53 inhibitors that compromise p53 function. Therefore, we hypothesized that oncolytic adenoviruses could be made more effective by suppressing p53 inhibitors in selected cancer cells. To investigate this concept, we attenuated the expression of the established p53 inhibitor synoviolin (SYVN1) in A549 lung cancer cells by RNA interference. Silencing *SYVN1* inhibited p53 degradation, thereby increasing p53 activity, and promoted adenovirus-induced A549 cell death. Based on these observations, we constructed a new oncolytic adenovirus that expresses a short hairpin RNA against *SYVN1*. This virus killed A549 cells more effectively in vitro and inhibited A549 xenograft tumor growth in vivo. Surprisingly, increased susceptibility to adenovirus-mediated cell killing by *SYVN1* silencing was also observed in A549 *TP53* knockout cells. Hence, while the mechanism of SYVN1-mediated inhibition of adenovirus replication is not fully understood, our results clearly show that RNA interference technology can be exploited to design more potent oncolytic adenoviruses.

## 1. Introduction

Oncolytic adenoviruses, which selectively replicate in cancer cells, represent a potential new treatment modality for solid tumors. Their capacity to kill cancer cells, evoking antitumor immune responses, and to produce viral progeny capable of spreading to neighboring tumor cells makes them particularly attractive anticancer agents. However, despite very encouraging results from in vitro and animal studies, the anticancer efficacy of oncolytic adenoviruses as a single agent in human clinical trials has been modest [1,2]. Thus, there is a clear need to increase the potency of oncolytic adenoviruses. One of the approaches taken to accomplish this is to arm the virus genome with a therapeutic transgene [3,4,5]. Increased potency was observed, e.g., by incorporating pro-apoptotic, fusogenic, or immune-modulating genes, or genes encoding prodrug-converting enzymes. One successful strategy to empower oncolytic adenoviruses has been to express the *TP53* tumor suppressor gene [6,7]. Exogenous p53 expression augmented oncolytic adenovirus replication in the majority of cancer cell lines, primary tumor cell cultures, and xenograft tumors in animal models [6,7,8,9].

Approximately 50% of all tumors retain wild-type p53 [10]. In many of these cancers, p53 function is hampered by one or more p53 inhibitors affecting p53 stability, localization, or transcriptional activity. Ubiquitin ligases represent an important class of p53 regulators, with MDM2 being the paradigm example [11]. We hypothesized, therefore, that oncolytic adenoviruses could potentially be made more effective by suppressing p53 inhibitors in cancer cells. Previously, we successfully silenced a reporter gene in cancer cells by incorporating an RNA interference (RNAi) cassette into the genome of an oncolytic adenovirus [12]. We reasoned, therefore, that RNAi technology could be used to design such more potent oncolytic adenoviruses. In this approach, specific RNAi-enhanced oncolytic viruses should be matched to tumor types with targeted p53 inhibitor expression. Silencing the identified inhibitor should increase p53 activity, thereby increasing oncolytic potency. In the present study, we tested this concept using the p53 wild-type A549 non-small cell lung cancer (NSCLC) cell line. We chose A549 cells because we previously completed genome-wide RNAi screens for modulators of p53 activity in this cell line [13]. In these studies, silencing the *SYVN1* gene reproducibly activated p53. *SYVN1*, also known as *HRD1* or *DER3*, encodes the endoplasmatic reticulum (ER)-resident E3 ubiquitin ligase synoviolin. Synoviolin is overexpressed in synoviocytes of patients with rheumatoid arthritis [14] and sequesters p53 in the cytoplasm to promote its degradation [15]. Thus, *SYVN1* is a confirmed target in A549 cells with a known mode of p53 inhibition.

Here we show that silencing *SYVN1* empowered adenovirus-mediated killing of A549 cells in vitro and in vivo. Surprisingly, however, this potency enhancement was also observed in *TP53* knockout A549 cells, demonstrating that the effect was at least partially p53-independent. Thus, the molecular mechanism of promotion of oncolysis by silencing *SYVN1* remains to be resolved. Our observations do, however, suggest that treatment of certain cancers with oncolytic adenoviruses could be made more effective by silencing *SYVN1* and confirm the utility of combining oncolytic adenoviruses with RNAi.

## 2. Results

### 2.1. Synoviolin Silencing Induces p53 Activity in A549 Lung Cancer Cells

**Figure 1:**
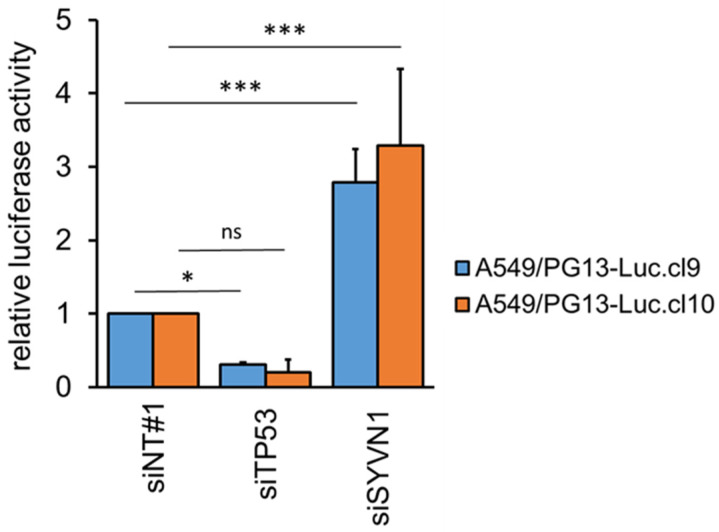
*SYVN1* silencing induces p53 activity in A549 cells. P53 activation upon gene silencing was determined in two independent A549-derivative cell lines carrying p53 reporter construct PG13-Luc. Cells were transfected with 50 nM small interfering RNA (siRNA) against *TP53* or *SYVN1* or irrelevant control siRNA. Seventy-two hours later, luciferase expression was measured. Blue and orange bars indicate results obtained on the two different A549/PG13-Luc reporter cell lines. Results shown are the mean fold induction in luciferase activity over negative control siRNA (NT#1) + SD of three or four independent experiments done in triplicate on A549/PG13-Luc.cl9 and A549/PG13-Luc.cl10 cells, respectively. Differences were tested using one-way ANOVA with Dunn’s multiple comparisons test. ns, not significant; *, *p* < 0.05; ***, *p* < 0.001.

We first confirmed functional inhibition of p53 by synoviolin in A549 lung adenocarcinoma cells. To this end, two independent A549/PG13-Luc reporter cell clones [16] that carry the plasmid PG13-Luc, containing 13 copies of the p53 binding consensus sequence upstream of a firefly luciferase reporter gene, were transfected with small interfering RNAs (siRNAs) targeting *SYVN1* or *TP53*, and p53 transcriptional activity was determined by measuring firefly luciferase activity (Figure 1). In addition to *SYVN1*, 16 other known p53 inhibitors were tested (Supplementary Figure S1). As expected, silencing *TP53* reduced p53 activity effectively. In contrast, higher p53 activity levels were observed upon silencing *SYVN1*. In the two independent A549/PG13-Luc reporter clones, silencing of *SYVN1* resulted on average in a 3-fold increase in p53 activity (*p* < 0.001). While silencing of some of the other p53 inhibitor genes also elevated p53 activity, silencing *SYVN1* stood out (Supplementary Figure S1). Thus, while multiple p53 inhibitors are active in A549 cells [13], *SYVN1* silencing alone was sufficient to significantly and reproducibly activate p53.

**Figure 2:**
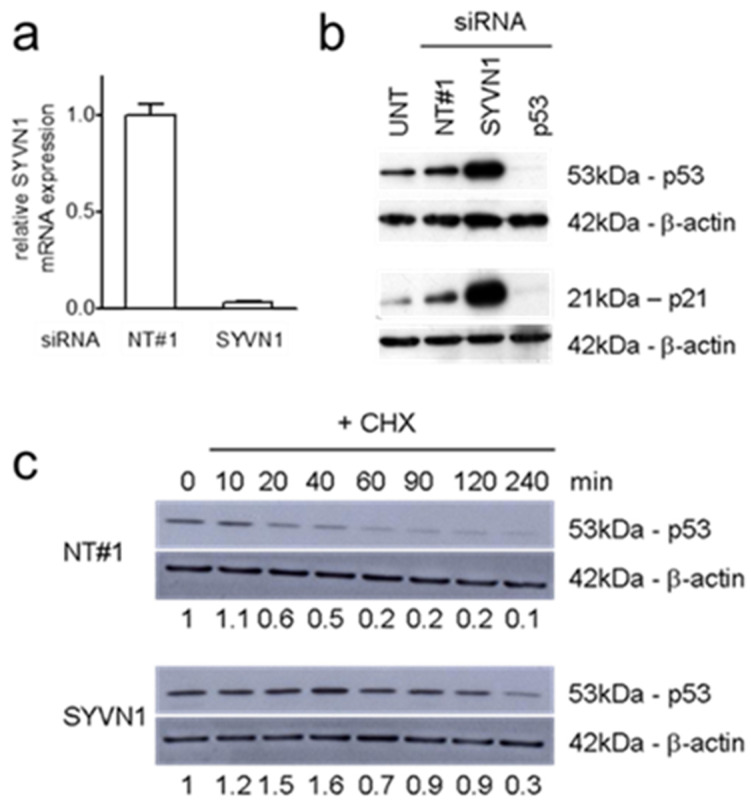
Silencing SYVN1 in A549 cells inhibits p53 degradation. (**a**) Quantification of SYVN1 mRNA in A549 cells transfected with negative control siRNA NT#1 or SYVN1 siRNA as indicated. The SYVN1 mRNA level was assessed by RT-qPCR and normalized to GAPDH mRNA. (**b**) SYVN1 silencing in A549 cells increases p53 protein levels (upper panels) and causes functional activation of p53, as evidenced by enhanced p21 expression (lower panels), determined by Western Blot analysis 96 h after siRNA transfection. siRNA against TP53 is included as control, eliciting an opposite effect. UNT, untransfected control cells. (**c**) SYVN1 silencing increases p53 half-life. Western blot analysis for p53 and β-actin was done at the indicated time points after siRNA transfection in the presence of cycloheximide (CHX). The relative fraction remaining p53 compared to *t* = 0 as quantified by densitometry and normalized to β-actin is given below the panels.

Synoviolin was previously described to sequester p53 in the cytoplasm and to promote its degradation [15]. Figure 2 shows that *SYVN1* silencing in A549 cells resulted in functional activation of p53 according to the mode of action described for synoviolin. RT-qPCR analysis showed that transfection of *SYVN1* siRNA very efficiently silenced *SYVN1* mRNA (Figure 2a). This caused an accumulation of p53 protein and enhanced expression of p21, a paradigm p53 transcriptional target gene (Figure 2b). Silencing *SYVN1* substantially prolonged the p53 half-life from approximately 30 min to more than 2 h (Figure 2c). Hence, in A549 cells, silencing *SYVN1* increases p53 transcriptional activity by inhibiting synoviolin-mediated p53 degradation.

### 2.2. Silencing Synoviolin Increases Oncolysis of Adenovirus-Infected A549 Cells

**Figure 3:**
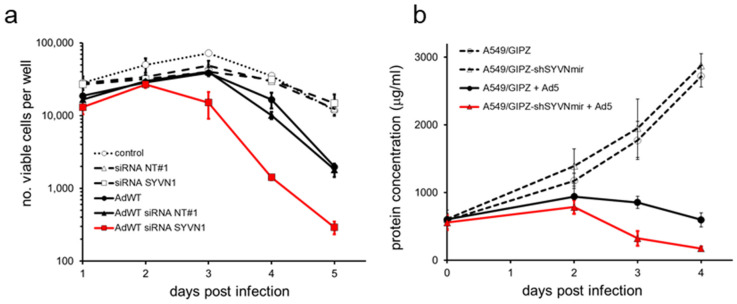
SYVN1 silencing decreases viability of Ad5-infected A549 cells. (**a**) A549 cells were transfected with SYVN1 siRNA or NT#1 siRNA and infected with 100 infectious units (IU)/cell Ad5. Viable cells were counted on days 1 to 5 after infection. Data shown are the mean ± SD of two independent experiments performed in triplicate. (**b**) A549 cells stably transfected with SYVN1-silencing lentiviral vector plasmid GIPZ-shSYVN1mir or with a GIPZ empty vector control plasmid were infected with 100 IU/cell Ad5. Cell viability was determined by BCA protein assay on day 0 and days 2 to 4 after infection. Data shown are the mean ± SD of four independent experiments.

Functional identification of p53 inhibitor synoviolin in A549 cells allowed us to investigate our hypothesis that reactivating endogenous p53 through silencing a p53 inhibitor using RNAi could enhance adenovirus propagation and oncolytic effect in cancer cells. We transfected A549 cells with *siSYVN1* or control siRNA and subjected the cells to infection with wild-type adenovirus serotype 5 (Ad5) or mock treatment. Subsequently, we counted viable cells microscopically 1 to 5 days after infection (Figure 3a). Cell killing by Ad5 became detectable 4 days after infection. Control siRNA or *SYVN1* siRNA treatment alone did not affect viable cell numbers. In contrast, silencing *SYVN1*, but not irrelevant control siRNA, reproducibly and selectively increased adenovirus cancer-cell-killing potency. Cell death became apparent 3 days after Ad5 infection in *SYVN1* siRNA-transfected cells, and cell viability was significantly lower in *SYVN1* siRNA-transfected cells than in control siRNA-transfected cells at days 3 to 5 after Ad5 infection (two-tailed *t*-test: all; *p* < 0.05).

We validated this finding independently using a GIPZ lentiviral vector expressing a short hairpin molecule designed to mimic a natural primary MicroRNA transcript (shRNAmir) directed against a target sequence on the *SYVN1* mRNA distinct from the sequences targeted by the siRNAs. A549 cells were stably transfected with the GIPZ-shSYVN1mir vector or with a GIPZ lentiviral empty vector control. *SYVN1* expression was silenced by 60% in A549/GIPZ-shSYVN1mir cells, as determined by RT-qPCR. A549/GIPZ and A549/GIPZ-shSYVN1mir cells were subjected to Ad5 infection, and 2 to 4 days later, in vitro cytotoxicity was determined by measuring total protein content of the adherent cell fraction. Stable *SYVN1* silencing alone did not affect cell viability. In contrast, it substantially reduced the number of adherent cells after Ad5 infection (Figure 3b). At 3 and 4 days after infection, Ad5 was significantly more cytotoxic to A549/GIPZ-shSYVN1mir cells than to A549/GIPZ cells (two-tailed *t*-test: *p* < 0.001).

### 2.3. Construction and Characterization of an Oncolytic Adenovirus Expressing a Short Hairpin RNA against SYVN1

Recently, we presented a platform for rapid production of replication-competent oncolytic adenoviruses expressing short hairpin RNA (shRNA) [17]. This platform uses a plasmid carrying a full length adenovirus genome, comprising the E1AΔ24 mutation [18], providing conditional replication property, and a Gateway destination cassette between the adenovirus E4 region and the right-hand ITR. A U6 promoter-driven shRNA targeting *SYVN1* (same stem–loop hairpin sequence as in GIPZ-shSYVN1mir) was inserted into this construct through a single in vitro recombination step, creating AdΔ24E3-U6.shSYVN1. AdΔ24E3-U6 [17] carrying an empty expression cassette served as control.

**Figure 4:**
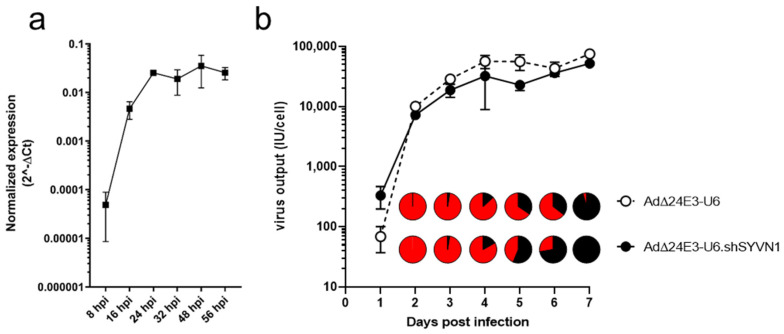
Characterization of oncolytic adenovirus-silencing SYVN1. (**a**) Expression and processing of adenovirus-encoded SYVN1 shRNA in cancer cells. A549 cells were infected with AdΔ24E3-U6 or AdΔ24E3-U6.shSYVN1 at 100 IU/cell, and total RNA was isolated at the indicated hours after infection. RT-qPCR was done for mature SYVN1 siRNA and for U6 small nuclear RNA as control. SYVN1 siRNA expression was determined using the ΔCt method. The graph shows mean mature SYVN1 siRNA expression in cells infected with AdΔ24E3-U6.shSYVN1 from three independent experiments, each performed in duplicate. SYVN1 siRNA expression was not detected in AdΔ24E3-U6-infected cells at any time point. (**b**) Effect of SYVN1 silencing on adenovirus propagation. A549 cells were infected with 10 IU/cell AdΔ24E3-U6 or AdΔ24E3-U6.shSYVN1. One to 7 days later, virus titers were determined in cells and culture medium separately. Lines show total infectious virus output in cultures infected with AdΔ24E3-U6 (broken line, open symbols) or AdΔ24E3-U6.shSYVN1 (continuous line, closed symbols). Pie chart inserts show the percentage infectious virus released in the culture medium (black sector) relative to the total amount infectious virus produced, measured at 2–7 days post-infection. Results are the means of an experiment performed in triplicate. Differences between the two viruses are not significant, except for total virus production on day 1 (*p* = 0.03, two-tailed *t*-test).

AdΔ24E3-U6.shSYVN1 was characterized for expression of mature *SYVN1* siRNA in infected cancer cells. To this end, A549/PG13-Luc cells were infected with AdΔ24E3-U6 or AdΔ24E3-U6.shSYVN1. At various time points after infection, mature siRNA expression was detected by RT-qPCR. *SYVN1* siRNA was produced in cells infected with AdΔ24E3-U6.shSYVN1 over the first 24 h after infection; thereafter, it remained present for at least more than a day (Figure 4a). Hence, oncolytic adenovirus-encoded *shSYVN1* was expressed during virus replication and processed by the RNAi machinery into mature siRNA.

Enhanced killing of adenovirus-infected cancer cells due to *SYVN1* silencing could perhaps interfere with virus propagation, if cell death is induced before the production of infectious progeny virus is completed. Alternatively, augmented cell death could accelerate progeny virus release. To test for these possibilities, A549 cells were infected with AdΔ24E3-U6 or AdΔ24E3-U6.shSYVN1, and virus titers were determined in cells and supernatant daily until one week after infection (Figure 4b). The two viruses exhibited similar virus production characteristics, although progeny virus titers appeared to increase more rapidly in AdΔ24E3-U6.shSYVN1-infected cells. On day 1, these cells exhibited an almost 5-fold higher titer (two-tailed *t*-test: *p* = 0.03). At all other time points, however, AdΔ24E3-U6.shSYVN1-infected cells produced slightly lower titers (1.2–2.4-fold; not significant). For both viruses, production was completed by day 4, at which time infected cells started to release substantial amounts of progeny virus. AdΔ24E3-U6.shSYVN1-infected cells released >50% of the progeny virus within 5 days, and release was complete after 7 days. Release of AdΔ24E3-U6 progeny seemed to lag behind, with only 35% released after 6 days. However, in absolute amounts, the difference in progeny release between the two viruses was not significant. Hence, induction of cell death by the combined action of adenovirus replication and *SYVN1* silencing had modest effects on the kinetics of infectious adenovirus progeny production and release.

### 2.4. Oncolytic Adenovirus Expressing a Short Hairpin RNA against SYVN1 Exhibits Enhanced Oncolysis In Vitro and In Vivo

**Figure 5:**
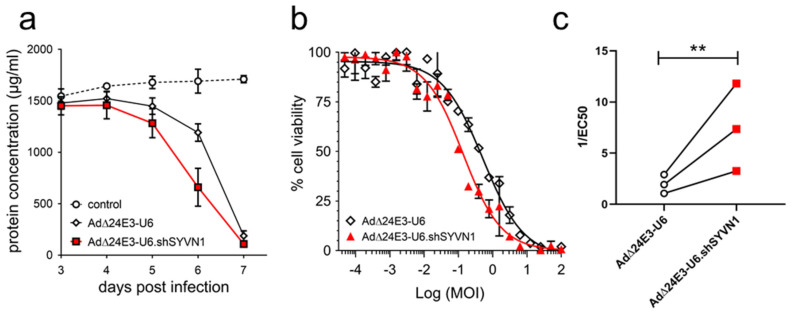
Oncolytic adenovirus expressing shRNA against *SYVN1* exhibits enhanced oncolysis in A549 cells. A549 cells were infected with AdΔ24E3-U6.shSYVN1 or AdΔ24E3-U6 at 10 IU/cell (**a**) or in a 2-fold dilution titration over the range of 4.8 × 10^−4^ to 100 IU/cell (**b**,**c**). Cell viability was determined by BCA protein assay on days 3 to 7 (**a**) or on day 7 (**b**,**c**) after infection. Both types of experiments were done three times in triplicate. Data in **a** are the mean results of three independent experiments ± SD; (**b**) shows representative sigmoidal dose-response curves from a single experiment in triplicate. [The two other experiments are shown in Supplementary Figure S2]; (**c**) shows the paired comparison of the oncolytic adenovirus cytotoxicity, i.e., 1/EC_50_ calculated from the dose-response curve of the two viruses in the three independent experiments. **, *p* < 0.01 (two-tailed paired *t*-test).

To investigate if oncolytic adenovirus-encoded *shSYVN1* could sensitize cancer cells to oncolysis, A549 cells were infected with AdΔ24E3-U6.shSYVN1 or AdΔ24E3-U6, and cytotoxicity was determined by measuring total protein content of the adherent cell fraction (Figure 5). To assess the time course of oncolysis, cells were infected at 10 IU/cell, and cytotoxicity was measured 3 to 7 days post-infection. The oncolytic adenovirus expressing an shRNA against *SYVN1* killed A549 cells more rapidly (Figure 5a). At 6 days after infection, AdΔ24E3-U6.shSYVN1 exhibited more cytotoxicity than AdΔ24E3-U6 (two-tailed *t*-test: *p* = 0.01). Next, to compare oncolytic propagation capacities, A549 cells were subjected to a dose range virus titration, and cytotoxicity was determined after 7 days. Dose-response curves were made (representative example in Figure 5b), and the virus dose required to reduce cell viability by 50% (EC_50_) was calculated as a measure for oncolytic activity, defined as 1/EC_50_. In three independent experiments, AdΔ24E3-U6.shSYVN1 exhibited an average 3.6 ± 0.07 SD higher oncolytic potency than AdΔ24E3-U6 (two-tailed paired *t*-test: *p* < 0.01) (Figure 5c).

**Figure 6:**
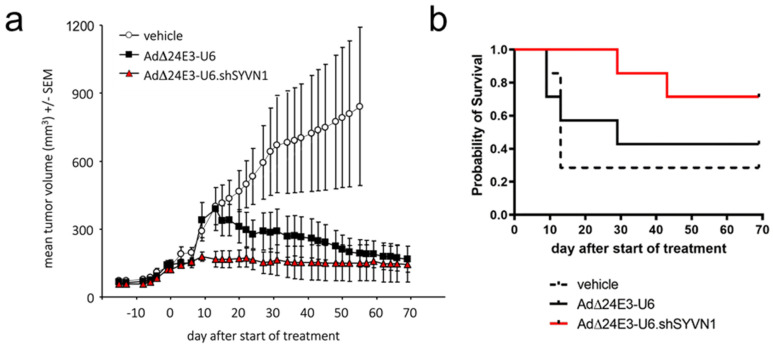
Antitumor activity of AdΔ24E3-U6 and AdΔ24E3-U6.shSYVN1 in subcutaneous A549 xenograft tumors in vivo. Mice bearing established A549 tumors were injected for 4 consecutive days with AdΔ24E3-U6 or AdΔ24E3-U6.shSYVN1 at a dose of 2.5 × 10^8^ IU or with vehicle. (**a**) Tumor sizes were measured for two months or until mice were sacrificed when tumors grew larger than 2000 mm^3^. Tumor volumes are given as mean ± SEM (*n* = 7). (**b**) Kaplan–Meier graph depicting the survival of mice with a tumor volume smaller than 3 times the tumor volume at start of treatment.

Next, we investigated oncolytic activities of AdΔ24E3-U6 and AdΔ24E3-U6.shSYVN1 in intact tumors in vivo. To this end, A549 subcutaneous xenograft tumors were established on the flanks of nude mice. Virus or vehicle were injected intra-tumorally, and tumor volumes were monitored. Both viruses effectively inhibited tumor growth compared to vehicle controls (Figure 6a). Tumor volumes of vehicle-treated controls expanded 6.2-fold in 55 days, at which point animals from this group had to be sacrificed because their tumor size exceeded 2000 mm^3^. At this time, AdΔ24E3-U6- and AdΔ24E3-U6.shSYVN1-treated tumors were on average only 1.3- and 1.2-fold larger than at the time of treatment. Although differences between the two viruses were not significant, *SYVN1* silencing appeared to elicit a more immediate tumor growth inhibition. AdΔ24E3-U6-induced tumor regression became apparent approximately 3 weeks after treatment but never reached significance due to a rather high tumor volume variability. In contrast, growth inhibition by AdΔ24E3-U6.shSYVN1 was evident already after 9 days and was significant from 2 to 5 weeks after treatment (*p* < 0.05, one-way ANOVA).

Ethical considerations precluded survival analysis. Therefore, the time until tumor volumes reached 3 times larger volumes compared to the volume at the start of treatment was used as a surrogate endpoint. This revealed a trend towards more effective tumor growth control by the *SYVN1*-silencing oncolytic adenovirus (Figure 6b). Five of the seven tumors in the AdΔ24E3-U6.shSYVN1 treatment group did not reach the endpoint over the course of the experiment, whereas only two and three tumors in the vehicle- and AdΔ24E3-U6-treated controls, respectively, did not pass the threshold. Median survivals were 13 days for vehicle controls and 29 days for AdΔ24E3-U6 treated mice, whereas for AdΔ24E3-U6.shSYVN1-treated animals, the median survival point was not reached. Due to the relatively small group sizes, however, these differences did not reach significance (*p* = 0.26, Log-rank Mantel–Cox test). Taken together, silencing *SYVN1* in oncolytic adenovirus-infected tumors seemed to bring about a more immediate and possibly more effective tumor growth control.

### 2.5. Enhanced Killing of Cancer Cells by SYVN1 Silencing Is p53-Independent

**Figure 7:**
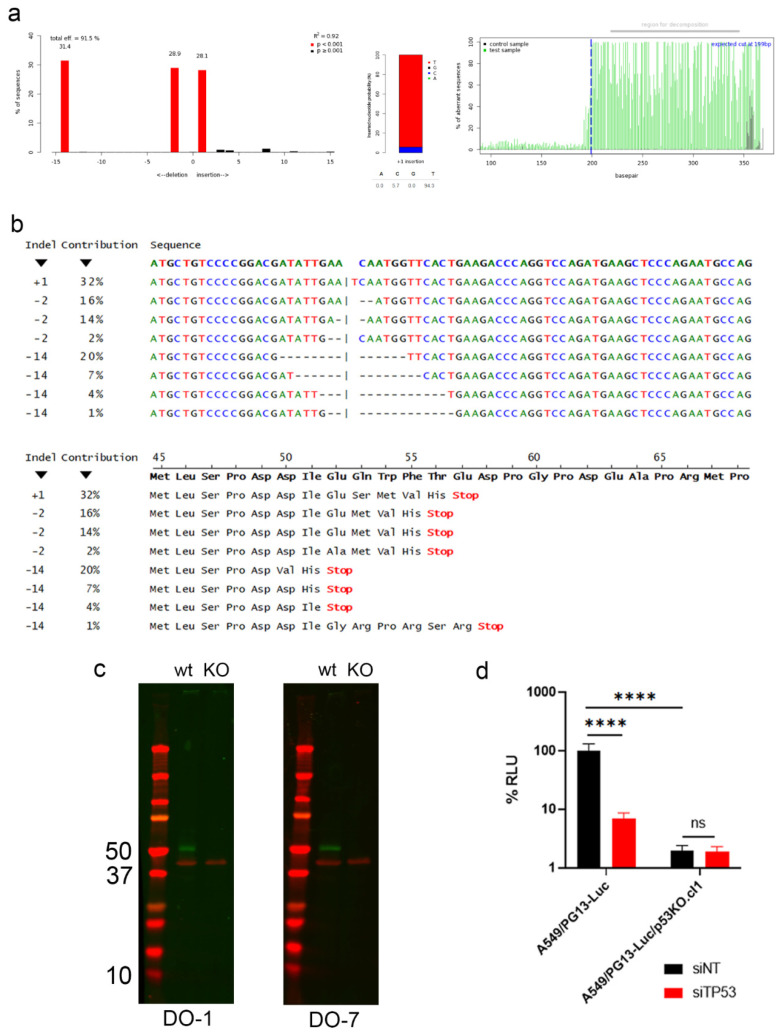
Characterization of *TP53* knockout cell line A549/PG13-Luc/p53KO.cl1. (**a**) TIDE analysis comparing *TP53* sequences of A549/PG13-Luc and A549/PG13-Luc/p53KO.cl1. From left to right, percent representation of different indels, showing the presence of −14, −2 and +1 indels in approximately equal proportions; probability of nucleotide insertion for the +1 indel, showing that this is likely a T; and quality control of the TIDE analysis, showing 100% divergence of the nucleotide sequence after the editing site. Pictures are from the analysis done using the coding strand sequencing results. Similar results (Supplementary Figure S3) were obtained after sequencing the complementary strand. (**b**) Predicted nucleotide and amino acid sequences according to ICE analysis. The first line shows the wild-type *TP53* sequence in bold. In the +1 indel, the N given by ICE was manually replaced by the T predicted by TIDE analysis. For each of the three indels, the predicted sequences are ranked by their probability. All predicted indels cause a frameshift creating a premature stop codon. They thus encode severely truncated proteins or will induce NMD of the transcript. (**c**) Western blot analysis of A549/PG13-Luc cells (lanes marked wt) and A549/PG13-Luc/p53KO.cl1 cells (lanes marked KO) using anti-p53 antibodies DO-1 and DO-7 (green). Both antibodies detect p53 protein in A549/PG13-Luc cells, but no (full-length or truncated) p53 protein in A549/PG13-Luc/p53KO.cl1 cells. Detection of β-actin (red) was done as loading control. Molecular weights of marker proteins (in kD) are indicated. (**d**) Luciferase expression by A549/PG13-Luc and A549/PG13-Luc/p53KO.cl1 cells, following transfection of 25 nM siNT#1 or *siTP53*. Data are given relative to the luminescence of A549/PG13-Luc cells transfected with siNT#1. The figure shows the mean + SD of an experiment done in triplicate. Significance was tested using one-way ANOVA with Tukey post-test for multiple comparisons. ns, not significant; ****, *p* < 0.0001.

To investigate if the oncolysis-enhancing effect of *SYVN1* silencing was caused by p53 activation or by an unsuspected p53-independent activity of synoviolin, we made a *TP53* knockout derivative of A549/PG13-Luc cells using CRISPR/Cas9 editing. Figure 7 describes the characterization of this A549/PG13-Luc/p53KO.cl1 cell line. Consistent with the hypotriploid karyotype of A549 cells, three distinct indels were identified in the *TP53* coding sequences (Figure 7a), each causing a predicted frameshift and a nonsense mutation near the editing site (Figure 7b). The nonsense mutations could result in the production of severely truncated dysfunctional p53 protein and/or in loss of p53 protein production by nonsense-mediated decay (NMD). With the editing site far upstream of the 3′ most exon–exon junction in the *TP53* mRNA, the latter is most likely to occur. To distinguish between the two options, we performed Western blot analysis with antibodies recognizing p53 epitopes mapping between amino acids encoded by *TP53* sequences 5′ of the editing site. To maximize the chance to detect truncated protein, we used two different antibodies, i.e., DO-1 recognizing a p53 epitope mapping between amino acids 11–25 and DO-7 recognizing a p53 epitope mapping between amino acids 1–45. As can be seen in Figure 7c, neither antibody detected a full-length or truncated low molecular weight protein in lysates of A549/PG13-Luc/p53KO.cl1 cells. Thus, editing caused NMD and complete depletion of p53 protein. Moreover, the cells exhibited strongly reduced luciferase expression from the PG13-Luc reporter construct (approximately 2% background p53-independent luminescence), which was unresponsive to transfection of siRNA-silencing *TP53*. In contrast, luciferase expressed by p53 wild-type A549/PG13-Luc cells was reduced more than 90% upon *siTP53* transfection (Figure 7d). Hence, A549/PG13 Luc/p53KO.cl1 cells are confirmed functional p53 knockout cells devoid of p53 protein and p53 transcriptional activity.

**Figure 8:**
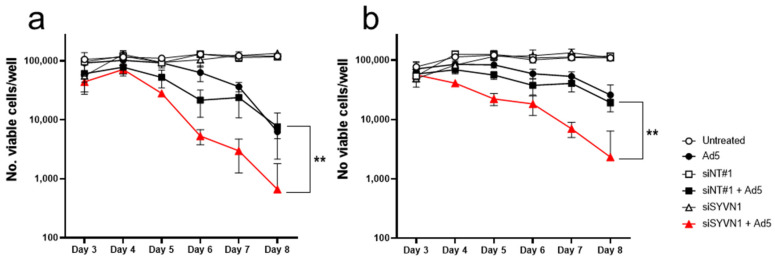
Analysis of the p53-dependency of oncolytic potency enhancement by silencing *SYVN1*. A549/PG13-Luc cells (**a**) and A549/PG13-Luc/p53KO.cl1 cells (**b**) were transfected with *siSYVN1* (triangles) or non-targeting control siNT#1 (squares) or left untransfected (circles) and infected one day later with Ad5 (closed symbols) or left uninfected (open symbols). Viable cell numbers are counted on days 3–8 after infection. Data shown are the mean ± SD of a typical experiment done in triplicate. **, *p* < 0.01.

A549/PG13-Luc and A549/PG13-Luc/p53KO.cl1 cells were transfected with siRNA-silencing *SYVN1* or with irrelevant control siRNA and infected with Ad5 the next day. Viable cell numbers were counted 3–8 days after infection (Figure 8). As can be seen, siRNA transfection did not influence cell viability, whereas Ad5 killed both cell lines, with cell numbers declining after day 5. The killing potency of Ad5 appeared reduced on the p53 knockout cell line compared to the isogenic control, which is in line with the known effect of p53 on adenovirus propagation. However, this difference did not reach significance. Importantly and unexpectedly, silencing *SYVN1* promoted cell killing by Ad5 compared to control siRNA on both cell lines. Over the course of the experiment, viability of Ad5/*siSYVN1*-treated cells was lower than that of Ad5/siNT-treated cells for both cell lines (*p* < 0.01, paired *t*-test). Hence, *SYVN1* silencing promoted Ad5-mediated A549 cell killing independent of functional p53.

## 3. Discussion

Results of several independent research groups have shown that functional tumor suppressor p53 expression in cancer cells augments the potency of oncolytic adenoviruses [6,7,19,20]. Based on this knowledge, development of p53-expressing oncolytic viruses is progressing, and their clinical utility is foreseen [21]. Nevertheless, while preclinical results suggest that a p53-expressing adenovirus should achieve stronger anticancer responses than previous-generation oncolytic adenoviruses, further improvements could be considered. In this respect, p53 inhibitor expression in cancer cells was found to reduce oncolytic adenovirus potency, as well as oncolytic adenovirus expressing wild-type p53 [22,23,24,25]. This inhibition could be negated either by expressing a p53 variant incapable of binding to the inhibitor [23,24,25] or by disrupting the p53–inhibitor interaction using a small molecule antagonist [22]. This led us to hypothesize that knowledge of p53 inhibitor expression in cancer cells could be exploited to design more effective oncolytic adenovirus therapies. To realize this, it should be assessed which p53 inhibitors are active in the cancer cells to be treated, and an effective means to suppress these p53 inhibitor activities should be developed. We envisioned that RNAi technology could be applied to accomplish both tasks. Functional screening for p53 activity upon silencing of putative p53 inhibitors in cancer cells using siRNA or shRNA molecules should identify active inhibitors, and the same molecules would then be used together with an oncolytic adenovirus to achieve effective cancer cell killing. The versatility of RNAi technology makes this approach potentially applicable to abolish oncolytic adenovirus potency inhibition by any p53 inhibitor.

Notably, we demonstrated previously that an shRNA expressed from the genome of an oncolytic adenovirus silenced its target gene during virus replication in human cancer cells effectively and specifically [12]. This offered the prospect of combining oncolytic adenoviruses and RNAi. Since then, there has been increasing interest in exploiting RNAi in the context of treating cancer with oncolytic adenoviruses [26]. Oncolytic adenoviruses were used as effective delivery platforms for shRNAs contributing to cell cycle arrest, apoptosis induction, or angiogenesis inhibition, together eliciting enhanced antitumor responses [27,28,29]. Here, we used RNAi technology to increase p53 stability and transcriptional activity in oncolytic adenovirus-infected cancer cells with the aim to boost oncolytic potency.

To test the concept, we chose the p53 wild-type A549 NSCLC cell line. Previously, we performed whole-genome siRNA library screening on this cell line and identified many target genes to activate p53 [13]. However, these functional screens did not distinguish between activating p53 by silencing genuine p53 inhibitors and induction of cellular stress responses that indirectly activate p53. Therefore, in the present study, we only included known p53 inhibitors. Among these, silencing *SYVN1* activated p53 in A549 cells most effectively. Although A549 cells express multiple p53 inhibitors, silencing *SYVN1* alone was sufficient to elevate p53 activity. *SYVN1* silencing in A549 cells could thus serve to test the concept of oncolytic adenovirus potency enhancement through endogenous p53 reactivation. As we had envisioned, silencing *SYVN1* did promote adenovirus-mediated A549 cell killing in vitro and A549 tumor growth inhibition in vivo.

Previously, exogenous p53 expression in adenovirus-infected cancer cells was found to enhance late adenoviral protein production and virus yield [30], accelerate cancer cell death and virus progeny release [7], and induce dual apoptotic and autophagic cell death in cancer cells [20]. Therefore, *SYVN1* silencing was assumed to elicit similar effects in adenovirus-infected A549 cells via activation of endogenous p53. In our experiments, *SYVN1* silencing alone did not have any appreciable effect on A549 cell viability. It did, however, promote the cytotoxic effects of adenovirus infection on both p53-proficient and p53-deficient A549 cells. Hence, while our observations do not formally exclude that synoviolin might have an effect on adenovirus replication via inhibition of p53 as well, they do show that synoviolin has at least a thus-far unrecognized p53-independent inhibitory effect on adenovirus replication in cancer cells. The underlying molecular mechanism remains to be resolved. Although this is beyond the scope of the present investigation, we hypothesize that perhaps the canonical anti-apoptotic function of synoviolin to protect cells against ER stress [31] plays a role. Interestingly, an RNAi screen for efficient cancer cell killing by a rhabdovirus identified several components of the ER stress response pathway, albeit not including SYVN1 [32]. Thus, inhibition of ER stress responses could be more widely applicable to promote the cytotoxicity of oncolytic viruses in cancer cells.

In cancer tissues, synoviolin expression is variable, with the highest expression (i.e., strong antibody staining) found in thyroid cancer, breast cancer, head and neck cancer, prostate cancer, and melanoma (www.proteinatlas.org (accessed in September 2022)). This suggests that oncolytic adenoviruses armed with a silencing cassette against *SYVN1* might be applicable for virotherapy of a variety of different cancers. Further studies in cancer cells with different synoviolin expression levels could perhaps shed more light on the molecular mechanism of its inhibitory effect on human adenoviruses.

From a general perspective, our findings show that oncolytic adenoviruses can be made more potent-cancer-cell killing agents by incorporating a silencing cassette against an inhibitor of adenovirus-mediated oncolysis. Adenovirus-encoded shRNA was properly expressed and processed by the RNAi machinery in the host cell. Mature siRNA duplexes were rapidly detected in infected cells, accumulating during the first day after infection. This expression was apparently sufficient to augment oncolytic potency. However, as we have recently shown, further improvements in the design of adenovirus-encoded gene-silencing cassettes are feasible. By expressing RNAi molecules not in an shRNA or precursor MicroRNA format but in a primary MicroRNA format, we achieved much higher expression levels [17]. In addition, improved siRNA expression and knockdown efficiency could also be sought by incorporating the results of studies that have defined shRNA sequence requirements for effective processing (e.g., [33,34]). While such modifications were apparently not necessary to detect functional consequences of silencing synoviolin in this study, they could be essential to elicit functional silencing effects on highly expressed proteins. For future development of potent RNAi-mediating oncolytic adenoviruses, it is thus recommended to incorporate these improvements.

## 4. Materials and Methods

### 4.1. Cell Lines and Culture Conditions

A549 lung adenocarcinoma cells were obtained from the American Type Culture Collection (Manassas, VA, USA). Cells were routinely grown at 37 °C and 5% CO_2_ in a humidified incubator in Dulbecco’s modified Eagle’s medium supplemented with 10% fetal bovine serum and antibiotics (100 U/mL penicillin and 100 μg/mL streptomycin).

A549/PG13-Luc cells expressing a p53-dependent reporter plasmid, comprising the firefly luciferase gene driven by a promoter containing thirteen p53-binding elements, were described before [16]. Two independent reporter cell lines were used, i.e., A549/PG13-Luc.cl9 [16] and A549/PG13-Luc.cl10. A549/PG13-Luc cells were maintained in medium supplemented with 400 μg/mL Hygromycin B (Roche #10843555001). A549/PG13-Luc derivative cells with complete knockout of the *TP53* gene were made and characterized as follows. A549/PG13-Luc.cl9 cells were co-transfected with pX330-Delta_Puro [35] in which single guide RNA sequence 5′-CCCCGGACGATATTGAACAA TGG-3′ targeting nt 137–153 of the human *TP53* open reading frame was inserted, as described by Ran et al. [36], along with pX330-U6-Chimeric_BB-CBh-hSpCas9 ([37]; a gift from Feng Zhang; Addgene plasmid # 42230), using Lipofectamine 3000 (Invitrogen, Bleiswijk, The Netherlands), in Opti-MEM reduced serum medium (Gibco, Bleiswijk, The Netherlands). Transiently transfected cells were selected for 2 days in 1 μg/mL puromycin (Sigma-Aldrich, Zwijndrecht, The Netherlands), following which cells were cloned by single-cell plating. Clones exhibiting reduced luciferase expression indicative of *TP53* knockout were expanded and analyzed for indels in the *TP53* gene. To this end, DNA isolated from the cells was PCR-amplified using primers 5′-GAGACCTGTGGGAAGCGAAA-3′ and 5′-GCTGCCCTGGTAGGTTTTCT-3′ and sequenced bidirectionally using the same primers (custom DNA sequencing service performed at Eurofins Genomics, Ebersberg, Germany). Parental cells were included for comparison to the wild-type *TP53* sequence. Indel analysis was done in June 2020 using TIDE version 3.3.0 (tide.nki.nl; [38]) and ICE (www.synthego.com) tools. Functional loss of p53 activity was confirmed by measuring reduced luciferase activity and loss of responsiveness to inhibition of luciferase activity by *siTP53* transfection (see Section 4.3).

A549-derivative cells with silenced *SYVN1* expression were made by transfecting A549 cells with lentiviral vector construct GIPZ-shSYVN1mir expressing the *SYVN1* shRNAmir sequence 5′-TGCTGTTGACAGTGAGCGCGCTCACCATCTTCATCAAGTA TAGTGAAGCCACAGATGTATACTTGATGAAGATGGTGAGCATGCCTACTGCCTCGGA-3′ (ThermoFisher Scientific Open Biosystems, Huntsville, AL, USA) using Lipofectamine 2000 (Invitrogen) and selection with 2 μg/mL puromycin (Sigma-Aldrich). Control cells carrying GIPZ lentiviral empty vector (Thermo Fisher Scientific Open Biosystems) were made similarly.

### 4.2. Adenoviruses

Wild-type human adenovirus serotype 5 (Ad5) was kindly provided by Dr. R.C. Hoeben (Leiden University Medical Center, Leiden, The Netherlands). Oncolytic adenovirus AdΔ24E3-U6 carrying an empty shRNA expression cassette was made before [17], and new oncolytic adenovirus-AdΔ24E3-U6.shSYVN1-silencing *SYVN1* was made using analogous procedures [17]. In short, a shuttle vector pAdΔ24E3-DEST-R [17] was used that comprises the full-length Ad5-Δ24E3 [39] genome with flanking PacI restriction sites and that carries a Gateway recombination destination cassette between the adenovirus E4 region and the right-hand ITR. A *SYVN1* shRNA-encoding sequence targeting nucleotides 648 to 669 of the coding sequence of the *SYVN1* gene was introduced into entry clone pSHAG-1 [40] (generously provided by Dr. G.J. Hannon, Cold Spring Harbor Laboratory, Cold Spring Harbor, NY, USA) to create pSHAG-shSYVN1. To this end, two annealed synthetic oligonucleotides (i.e., 5′-GCTCACCATCTTCATCAAGTATAGTGAAGCCACAGATGTATACTTGATGAAGATGGTGAGCTTTTT-3′ and 5′-GATCAAAAAGCTCACCATCTTCATCAAGTATACATC TGTGGCTTCACTATACTTGATGAAGATGGTGAGCCG-3′; purchased from BaseClear, Leiden, The Netherlands) were introduced by ligation into pSHAG-1 digested with BseRI and BamHI. Next, the *SYVN1* shRNA-encoding U6 promoter-driven expression cassette from pSHAG-shSYVN1 was transferred into pAdΔ24E3-DEST-R via an LR GATEWAY in vitro recombination reaction using the GATEWAY LR Clonase enzyme mix (Invitrogen), according to the manufacturer’s protocol, to create pAdΔ24E3-U6.shSYVN1. Finally, the full-length AdΔ24-type oncolytic adenovirus genome with inserted *shSYVN1* expression cassette was released from pAdΔ24E3-U6.shSYVN1 by PacI digestion and transfected using lipofectamine reagent into A549 cells to obtain AdΔ24E3-U6.shSYVN1 virus. All viruses were propagated on A549 cells. The E1Δ24 deletion and the U6.shSYVN1 insertion were confirmed by PCR, and functional virus titers were determined by limiting-dilution titration and hexon staining using the AdenoX rapid titer kit (BD Biosciences, Breda, The Netherlands) according to standard techniques.

### 4.3. P53 Reporter Assays

A549/PG13-Luc or A549/PG13-Luc/p53KO.cl1 reporter cells were seeded 5000 cells per well in 96-well plates and transfected the next day with SMARTpool siRNA duplexes from Thermo Fisher Scientific Dharmacon (Lafayette, CO, USA), according to the manufacturer’s protocol, using 25 or 50 nM siRNA as indicated and 1000-times diluted Dharmafect 1 (Dharmacon). The SMARTpool siRNA reagents used were siCONTROL-1 (NT#1; #D-001210-01), *siTP53* (#M-003329-01), and *siSYVN1* (#M-007090-01). After 72 h, the culture medium was replaced by Luciferase Chemiluminescent Assay System Reporter Lysis Buffer (Promega, Madison, WI, USA), and the culture plates were subjected to a single freeze/thaw cycle. Chemiluminescence was measured with a Berthold Lumat LB 9507 luminometer during the 10 s immediately after addition of the cell lysate to the Luciferase Assay Reagent. Differences between *siTP53* or *siSYVN1* versus siNT#1-treated controls were tested by one-way ANOVA.

### 4.4. Quantitative Reverse Transcription Polymerase Chain Reaction

Cells from individual knockdown experiments were collected by centrifugation 96 h after siRNA transfection. RNA was prepared using the RNeasy kit (Qiagen, Hilden, Germany) according to the manufacturer’s protocol. Total RNA (1μg) was reverse transcribed using SuperScript III reverse transcriptase (Life Technologies, Bleiswijk, The Netherlands) after priming with random hexamers (Life Technologies). Real-time quantitative PCR was carried out on a Roche LS480 instrument in a 20 μL reaction containing 10 μL of SYBR Green PCR mix (Qiagen), diluted cDNA, and primers. QuantiTect Primers for human *SYVN1* (QT01669983) and *GAPDH* (QT01192646) were purchased from Qiagen. Relative mRNA levels compared to *GAPDH* controls were calculated using the ΔCt method.

Cells infected with 100 IU/cell AdΔ24E3-U6 or AdΔ24E3-U6.shSYVN1 were harvested at 8–56 h after infection, and RNA was isolated using the miRNeasy kit (Qiagen) according to the manufacturer’s protocol. Total RNA (200 ng) was reverse transcribed using SuperScript III reverse transcriptase and primer 5′-GTCGTATCC AGTGCAGGGTCCGAGGTATTCGCACTGGATACGACACTTGA-3′ for *SYVN1* siRNA or 5′-GTCATCCTTGCGCAGG-3′ for *U6* small nuclear RNA. Real-time quantitative PCR was done as above using primers 5′-GCCCGCCTCACCATCTTCATC-3′ and 5′-AGTGCAGGGTCCGAGGT-3′ for *SYVN1* siRNA or 5′-CGCTTCGGCAGCACATATAC-3′ and 5′-AGGGGCCATGCTAATCTTCT-3′ for *U6*, respectively. Oligonucleotide primers were purchased from Isogen Life Science (De Meern, The Netherlands). Relative *SYVN1* siRNA levels compared to *U6* controls were calculated using the ΔCt method.

### 4.5. Western Blot Analysis

Whole cell lysates prepared from A549 cells, 96 h after transfection with SMARTpool siRNA against human *SYVN1* or irrelevant negative control siRNA, were analyzed by PAGE and immunoblotting following established protocols. To study p53 protein half-life, translation was blocked by incubation with 10 mM cycloheximide (CHX; Sigma) for up to 4 h. Antibodies against human β-actin and p53 (clone DO-7) were obtained from DAKO (Glostrup, Denmark); anti-p21 antibody clone OP64 was obtained from Calbiochem (Merck, Darmstadt, Germany); goat and rabbit anti-mouse-HRP secondary antibody conjugates were from DAKO. Detection was done using ECL Plus substrate (GE Healthcare, Chicago, IL, USA), with the chemiluminescent signal captured by autoradiography. To detect p53 depletion by *TP53* knockout, cell lysates prepared from A549/PG13-Luc and A549/PG13-Luc/p53KO.cl1 cells were separated on a 4–20% Mini-PROTEAN TGX™ Precast Protein Gel (Bio-Rad Laboratories, Veenendaal, The Netherlands). Precision Plus Protein Dual Color Standard (Bio-Rad #1610374) was used as molecular weight marker. Proteins were transferred to Immobilon^®^-FL PVDF (Millipore; Merck Life Science, Amsterdam, The Netherlands) using a wet-transfer system. Membranes were first incubated with anti-human p53 MoAbs DO-1 (Santa Cruz Biotechnology, Dallas, TX, USA) and DO-7 (DAKO) and subsequently with IRDye^®^ 800CW Goat anti-mouse IgG (LI-COR Biosciences, Lincoln, NE, USA) secondary antibody. Thereafter, β-actin was detected on the same membranes using rabbit anti-human β-actin antibody (Bioké, Leiden, The Netherlands) and IRDye^®^ 680RD Goat anti-rabbit IgG antibody (LI-COR Biosciences). The blots were imaged on the Odyssey^®^ 9120 Infrared Imaging System (LI-COR Biosciences) in the 700 nm and 800 nm channels, and overlay pictures were made.

### 4.6. Cell Count Assay

Cells were seeded 5000 per well in a 96-well plate and cultured overnight. The next day, cells were transfected with 50 nM control or *SYVN1* siRNA using Dharmafect 1 as above or left untreated. Twenty-four hours post transfection, cells were infected with Ad5 at 100 IU/cell in a total volume of 150 μL or mock infected. On the indicated days after infection, cells were harvested by trypsinization. Cell suspensions were mixed 1:1 with 0.4% trypan blue solution (Sigma-Aldrich), and viable cells were counted using a cell counting chamber (Bürker hemacytometer). Results are the mean number of viable cells per well from triplicate wells of two individual experiments. Differences between siNT#1- and *siSYVN1*-treated cultures were tested by two-tailed paired *t*-test.

### 4.7. BCA Protein Assay

Cells were seeded and infected with Ad5, AdΔ24E3-U6.shSYVN1, or AdΔ24E3-U6 at the indicated multiplicity of infection (MOI). On the indicated days after infection, protein concentrations were determined using the BCA protein assay kit (Pierce, Rockford, IL, USA), according to the manufacturer’s protocol. Briefly, culture medium was replaced by 25 μL lysis buffer after washing with PBS. Cell lysates were mixed with BCA reagent, and plates were placed at 37 °C in a 5% CO_2_ incubator. After 30 min, 50 μL 3% SDS was added, and absorbance was measured at 595 nm using a Bio-Rad model microplate reader. Protein concentration (μg/mL) of each sample was calculated using a BSA standard curve. For dose-response calculations, sigmoidal curves were fit and EC50 values were calculated; differences between treatment groups compared in independent experiments were tested by paired *t*-test.

### 4.8. Adenovirus Production Assay

Cells were seeded 10^4^ per well in a 96-well plate and cultured overnight. The next day, cells were infected with AdΔ24E3-U6.shSYVN1 or AdΔ24E3-U6 at 10 IU/cell. Three hours after infection, the input virus was washed away. Cells were cultured in 200 μL medium for 1–7 days. At these time points, cell and supernatant fractions were collected separately, and virus was released by three freeze-thaw cycles. The supernatant fraction was collected by harvesting the upper 100 μL from the wells. This fraction was considered to contain 50% of the total amount of released virus. The remaining 100 μL culture medium with adherent, semi-adherent, and detached cells was collected as cell fraction. The amount of progeny virus inside the cells was calculated by subtracting the virus titer in the supernatant fraction from the titer in the cell fraction. The number of infectious virus particles was determined by subjecting A549 cells to a virus-limiting dilution titration in triplicate. After 48 h, cells were fixed in methanol and stained for hexon expression using the mouse anti-hexon antibody and rat anti-mouse immunoglobulin from the Adeno-X Rapid Titer Kit (Takara Bio, Shiga, Japan). The number of hexon-positive cells per well was counted, and virus titers were calculated.

### 4.9. Evaluation of Oncolytic Activity in Xenograft Tumors

The animal experiment was done according to guidelines established by the European community and procedures approved by the local scientific and ethical committees on animal experiments at the Cancer Center Amsterdam and VU University/VU University medical center (protocol codes CWO-DEC 10–55 and DEC-Onc-11-01A01). Six-to-eight-week-old female athymic nu/nu mice (purchased from Harlan Laboratories, Horst, The Netherlands) were allowed to acclimatize at the experimental location for at least one week. They were then injected subcutaneously on the flank with 10^7^ A549 cells and tumor size was monitored using a caliper. Tumor volumes were calculated according to the formula volume = (width)^2^ × length/2. Eighteen days after injection, mice bearing s.c. tumors of 85–240 mm^3^ (aim was 100–300 mm^3^) were randomly assigned to treatment groups (7 mice per group; group sizes were chosen based on a one-sided power analysis, power = 0.8, type I error *p* < 0.05, expected standard deviation 35%, and detectable effect size at least 50%) and injected intra-tumorally on 4 subsequent days with 50 μL AdΔ24E3-U6, AdΔ24E3-U6.shSYVN1, or vehicle (PBS/1% glycerol). Experimental group allocation was not blinded to the investigators. The first day of treatment was defined as day 0 of the experiment. The total cumulative virus dose given was 10^9^ IU. Tumor sizes were measured two or three times weekly for two months or until mice were sacrificed when tumor volume exceeded 2000 mm^3^. No data were excluded. Statistical difference in tumor size between treatment groups and controls was assessed at weekly intervals by one-way ANOVA with Tukey’s multiple comparison test; differences in survival were assessed by Log-rank Mantel–Cox test.

### 4.10. Statistical Analysis

All statistical analyses, using the tests indicated with the description of the experiments, were done using GraphPad Prism version 8.2.1 or 9.3.1 (GraphPad Software, San Diego, CA, USA).

## Data Availability

Not applicable.

## Supplementary Materials

The following supporting information can be downloaded at: https://www.mdpi.com/article/10.3390/ijms232315430/s1.

## References

[1] Chaurasiya S., Fong Y., Warner S.G. (2021). Oncolytic Virotherapy for Cancer: Clinical Experience. Biomedicines.

[2] Macedo N., Miller D.M., Haq R., Kaufman H.L. (2020). Clinical landscape of oncolytic virus research in 2020. J. Immunother. Cancer.

[3] Cody J.J., Douglas J.T. (2009). Armed replicating adenoviruses for cancer virotherapy. Cancer Gene Ther..

[4] de Gruijl T.D., Janssen A.B., van Beusechem V.W. (2015). Arming oncolytic viruses to leverage antitumor immunity. Expert Opin. Biol. Ther..

[5] Farrera-Sal M., Fillat C., Alemany R. (2020). Effect of Transgene Location, Transcriptional Control Elements and Transgene Features in Armed Oncolytic Adenoviruses. Cancers.

[6] Sauthoff H., Pipiya T., Heitner S., Chen S., Norman R.G., Rom W.N., Hay J.G. (2002). Late expression of p53 from a replicating adenovirus improves tumor cell killing and is more tumor cell specific than expression of the adenoviral death protein. Hum. Gene Ther..

[7] van Beusechem V.W., van den Doel P.B., Grill J., Pinedo H.M., Gerritsen W.R. (2002). Conditionally replicative adenovirus expressing p53 exhibits enhanced oncolytic potency. Cancer Res..

[8] Geoerger B., van Beusechem V.W., Opolon P., Morizet J., Laudani L., Lecluse Y., Barrois M., Idema S., Grill J., Gerritsen W.R. (2005). Expression of p53, or targeting towards EGFR, enhances the oncolytic potency of conditionally replicative adenovirus against neuroblastoma. J. Gene Med..

[9] Geoerger B., Vassal G., Opolon P., Dirven C.M., Morizet J., Laudani L., Grill J., Giaccone G., Vandertop W.P., Gerritsen W.R. (2004). Oncolytic activity of p53-expressing conditionally replicative adenovirus AdDelta24-p53 against human malignant glioma. Cancer Res..

[10] Vogelstein B., Lane D., Levine A.J. (2000). Surfing the p53 network. Nature.

[11] Lee J.T., Gu W. (2010). The multiple levels of regulation by p53 ubiquitination. Cell Death Differ..

[12] Carette J.E., Overmeer R.M., Schagen F.H., Alemany R., Barski O.A., Gerritsen W.R., Van Beusechem V.W. (2004). Conditionally replicating adenoviruses expressing short hairpin RNAs silence the expression of a target gene in cancer cells. Cancer Res..

[13] Siebring-van Olst E., Blijlevens M., de Menezes R.X., van der Meulen-Muileman I.H., Smit E.F., van Beusechem V.W. (2018). A genome-wide siRNA screen for regulators of tumor suppressor p53 activity in human non-small cell lung cancer cells identifies components of the RNA splicing machinery as targets for anticancer treatment. Mol. Oncol..

[14] Amano T., Yamasaki S., Yagishita N., Tsuchimochi K., Shin H., Kawahara K., Aratani S., Fujita H., Zhang L., Ikeda R. (2003). Synoviolin/Hrd1, an E3 ubiquitin ligase, as a novel pathogenic factor for arthropathy. Genes Dev..

[15] Yamasaki S., Yagishita N., Sasaki T., Nakazawa M., Kato Y., Yamadera T., Bae E., Toriyama S., Ikeda R., Zhang L. (2007). Cytoplasmic destruction of p53 by the endoplasmic reticulum-resident ubiquitin ligase ‘Synoviolin’. EMBO J..

[16] Siebring-van Olst E., Vermeulen C., de Menezes R.X., Howell M., Smit E.F., van Beusechem V.W. (2013). Affordable luciferase reporter assay for cell-based high-throughput screening. J. Biomol. Screen.

[17] Brachtlova T., van Ginkel J.W., Luinenburg M.J., de Menezes R.X., Koppers-Lalic D., Pegtel D.M., Dong W., de Gruijl T.D., van Beusechem V.W. (2020). Expression of Oncolytic Adenovirus-Encoded RNAi Molecules Is Most Effective in a pri-miRNA Precursor Format. Mol. Ther. Oncolytics.

[18] Fueyo J., Gomez-Manzano C., Alemany R., Lee P.S., McDonnell T.J., Mitlianga P., Shi Y.X., Levin V.A., Yung W.K., Kyritsis A.P. (2000). A mutant oncolytic adenovirus targeting the Rb pathway produces anti- glioma effect in vivo. Oncogene.

[19] Wang X., Su C., Cao H., Li K., Chen J., Jiang L., Zhang Q., Wu X., Jia X., Liu Y. (2008). A novel triple-regulated oncolytic adenovirus carrying p53 gene exerts potent antitumor efficacy on common human solid cancers. Mol. Cancer Ther..

[20] Hasei J., Sasaki T., Tazawa H., Osaki S., Yamakawa Y., Kunisada T., Yoshida A., Hashimoto Y., Onishi T., Uno F. (2013). Dual programmed cell death pathways induced by p53 transactivation overcome resistance to oncolytic adenovirus in human osteosarcoma cells. Mol. Cancer Ther..

[21] Bressy C., Hastie E., Grdzelishvili V.Z. (2017). Combining Oncolytic Virotherapy with p53 Tumor Suppressor Gene Therapy. Mol. Ther. Oncolytics.

[22] Graat H.C., Carette J.E., Schagen F.H., Vassilev L.T., Gerritsen W.R., Kaspers G.J., Wuisman P.I., van Beusechem V.W. (2007). Enhanced tumor cell kill by combined treatment with a small-molecule antagonist of mouse double minute 2 and adenoviruses encoding p53. Mol. Cancer Ther..

[23] Heideman D.A., Steenbergen R.D., van der Torre J., Scheffner M., Alemany R., Gerritsen W.R., Meijer C.J., Snijders P.J., van Beusechem V.W. (2005). Oncolytic adenovirus expressing a p53 variant resistant to degradation by HPV E6 protein exhibits potent and selective replication in cervical cancer. Mol. Ther..

[24] Sauthoff H., Pipiya T., Chen S., Heitner S., Cheng J., Huang Y.Q., Rom W.N., Hay J.G. (2006). Modification of the p53 transgene of a replication-competent adenovirus prevents mdm2- and E1b-55kD-mediated degradation of p53. Cancer Gene Ther..

[25] van Beusechem V.W., van den Doel P.B., Gerritsen W.R. (2005). Conditionally replicative adenovirus expressing degradation-resistant p53 for enhanced oncolysis of human cancer cells overexpressing murine double minute 2. Mol. Cancer Ther..

[26] Brachtlova T., van Beusechem V.W. (2018). Unleashing the Full Potential of Oncolytic Adenoviruses against Cancer by Applying RNA Interference: The Force Awakens. Cells.

[27] Yoo J.Y., Kim J.H., Kwon Y.G., Kim E.C., Kim N.K., Choi H.J., Yun C.O. (2007). VEGF-specific short hairpin RNA-expressing oncolytic adenovirus elicits potent inhibition of angiogenesis and tumor growth. Mol. Ther..

[28] Zhang Y.A., Nemunaitis J., Samuel S.K., Chen P., Shen Y., Tong A.W. (2006). Antitumor activity of an oncolytic adenovirus-delivered oncogene small interfering RNA. Cancer Res..

[29] Zheng J.N., Pei D.S., Sun F.H., Zhang B.F., Liu X.Y., Gu J.F., Liu Y.H., Hu X.L., Mao L.J., Wen R.M. (2009). Inhibition of renal cancer cell growth by oncolytic adenovirus armed short hairpin RNA targeting hTERT gene. Cancer Biol. Ther..

[30] Royds J.A., Hibma M., Dix B.R., Hananeia L., Russell I.A., Wiles A., Wynford-Thomas D., Braithwaite A.W. (2006). p53 promotes adenoviral replication and increases late viral gene expression. Oncogene.

[31] Yamasaki S., Yagishita N., Nishioka K., Nakajima T. (2007). The roles of synoviolin in crosstalk between endoplasmic reticulum stress-induced apoptosis and p53 pathway. Cell Cycle.

[32] Mahoney D.J., Lefebvre C., Allan K., Brun J., Sanaei C.A., Baird S., Pearce N., Gronberg S., Wilson B., Prakesh M. (2011). Virus-tumor interactome screen reveals ER stress response can reprogram resistant cancers for oncolytic virus-triggered caspase-2 cell death. Cancer Cell.

[33] Fellmann C., Zuber J., McJunkin K., Chang K., Malone C.D., Dickins R.A., Xu Q., Hengartner M.O., Elledge S.J., Hannon G.J. (2011). Functional identification of optimized RNAi triggers using a massively parallel sensor assay. Mol. Cell.

[34] Gu S., Jin L., Zhang Y., Huang Y., Zhang F., Valdmanis P.N., Kay M.A. (2012). The loop position of shRNAs and pre-miRNAs is critical for the accuracy of dicer processing in vivo. Cell.

[35] Blanas A., Cornelissen L.A.M., Kotsias M., van der Horst J.C., van de Vrugt H.J., Kalay H., Spencer D.I.R., Kozak R.P., van Vliet S.J. (2019). Transcriptional activation of fucosyltransferase (FUT) genes using the CRISPR-dCas9-VPR technology reveals potent N-glycome alterations in colorectal cancer cells. Glycobiology.

[36] Ran F.A., Hsu P.D., Wright J., Agarwala V., Scott D.A., Zhang F. (2013). Genome engineering using the CRISPR-Cas9 system. Nat. Protoc..

[37] Cong L., Ran F.A., Cox D., Lin S., Barretto R., Habib N., Hsu P.D., Wu X., Jiang W., Marraffini L.A. (2013). Multiplex genome engineering using CRISPR/Cas systems. Science.

[38] Brinkman E.K., Chen T., Amendola M., van Steensel B. (2014). Easy quantitative assessment of genome editing by sequence trace decomposition. Nucleic Acids Res..

[39] Suzuki K., Alemany R., Yamamoto M., Curiel D.T. (2002). The presence of the adenovirus E3 region improves the oncolytic potency of conditionally replicative adenoviruses. Clin. Cancer Res..

[40] Paddison P.J., Caudy A.A., Bernstein E., Hannon G.J., Conklin D.S. (2002). Short hairpin RNAs (shRNAs) induce sequence-specific silencing in mammalian cells. Genes Dev..

