# Supplementary material for: Evaluation of a Novel Oncolytic Adenovirus Silencing SYVN1
Source: Int J Mol Sci. 2022 Dec 6;23(23):15430. doi: 10.3390/ijms232315430 (PMC9737683; doi:10.3390/ijms232315430)
Supplement: Supplementary file 1 [file ijms-23-15430-s001.zip › ijms-1980610-supplementary.pdf]

Supplementary Figures and Table

Vermeulen, C.; Brachtlova, T.; Tol, N.; van der Meulen-Muileman, I.H.; Hodzic, J.; van de Vrugt, H.J.; van Beusechem, V.W. *Evaluation of a novel oncolytic adenovirus silencing SYVN1.*

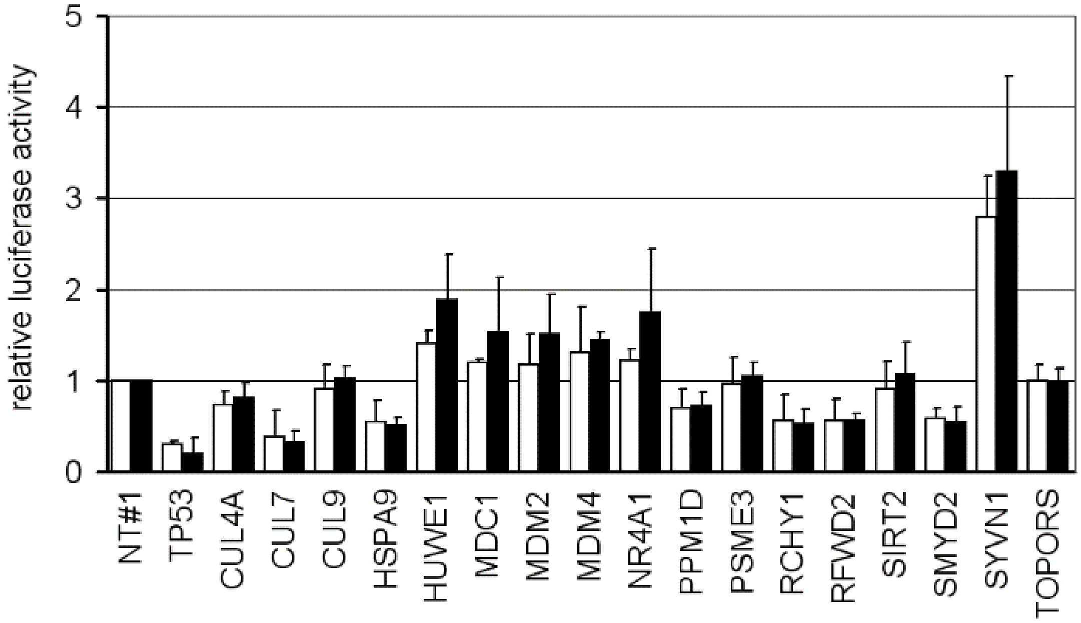

Figure S1. Miniscreen for inhibitors of p53 activity in A549 NSCLC cells. P53 activation upon silencing of indicated genes was determined in two independent A549 derivative cell lines carrying p53 reporter construct PG13-Luc. Cells were transfected with 50 nM siRNAs against p53,17 putative p53 inhibitors and irrelevant control siRNA. Details on the siRNA reagents used are given in Supplementary Table 1. Seventy-two hours later, luciferase expression was measured. Results shown are the mean fold induction in luciferase activity over negative control siRNA (NT#1) + SD of 3 or 4 independent experiments done in triplicate on A549/PG13-Luc.cl9 (white bars) and A549/PG13-Luc.cl10 (black bars) cells, respectively. Results with siNT#1, *siTP53* and *siSYVN1* are also shown in Figure1.

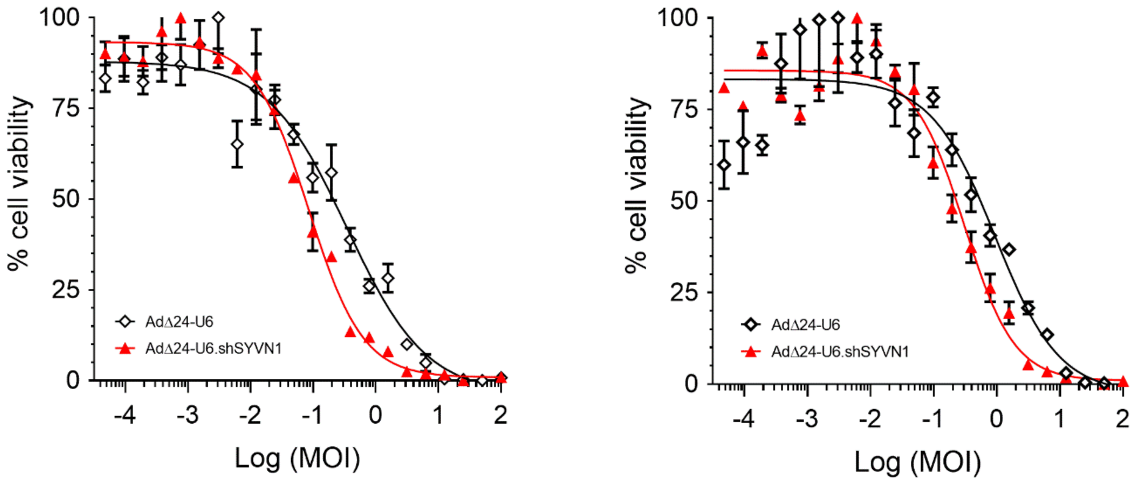

Figure S2. A549 cell viability 7 days after infection with AdΔ24E3-U6.shSYVN1 or AdΔ24E3-U6 at 10 IU/cell, as determined by BCA protein assay. Three independent experiments were done in triplicate. One representative experiment is shown in Figure 5b. This figure shows the two other experiments. The combined results of the 3 independent experiments were used to compile Figure 5c.

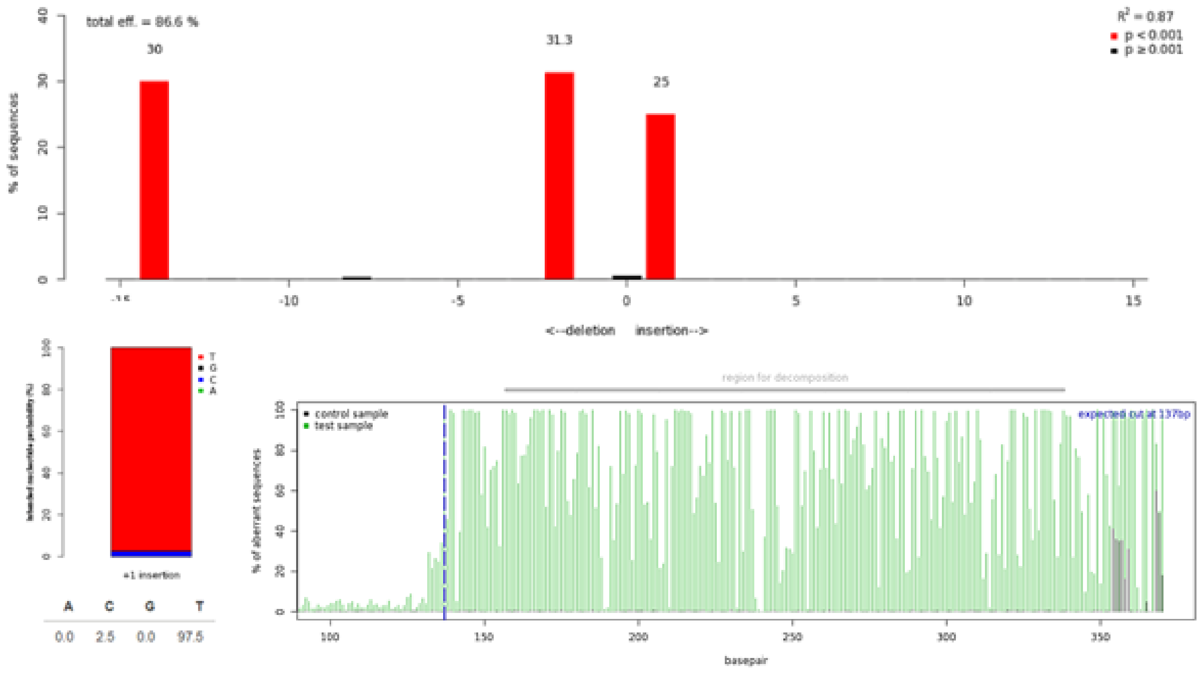

Figure S3. TIDE analysis comparing *TP53* sequences of A549/PG13-Luc and A549/PG13-Luc/p53KO.cl1, using the complementary strand sequencing results. The analysis confirms the indel identification based on the coding sequence analysis shown in Figure 7.

Table S1. siRNA SMARTpool reagents used to identify p53 inhibitor genes in A549 cells.

human target gene	Gene accession number	siRNA SMARTpool catalog number
siCONTROL-1 (NT#1)		D-001210-01
CUL4A	NM_001008895	M-012610-01
CUL7	NM_014780	M-017673-00
CUL9, PARC	NM_015089	M-014128-00
HSPA9	NM_004134	M-004750-03
HUWE1	NM_031407	M-007185-01
MDC1	NM_014641	M-003506-04
MDM2	NM_002392	M-003279-02
MDM4	NM_002393	M-006536-03
NR4A1	NM_002135	M-003426-03
PPM1D	NM_003620	M-004554-00
PSME3	NM_005789	M-012133-00
RCHY1, PIRH2	NM_015436	M-006966-00
RFWD2, COP1	NM_001001740	M-007049-00
SIRT2	NM_012237	M-004826-02
SMYD2	NM_020197	M-020291-00
SYVN1	NM_032431	M-007090-01
TOPORS	NM_005802	M-020048-00
TP53	NM_000546	M-003329-01
